# Supplementary material for: TiMEG: an integrative statistical method for partially missing multi-omics data
Source: Sci Rep. 2021 Dec 15;11:24077. doi: 10.1038/s41598-021-03034-z (PMC8674330; doi:10.1038/s41598-021-03034-z)
Supplement: Supplementary file 1 — Supplementary Information 1. [file 41598_2021_3034_MOESM1_ESM.pdf]

TiMEG: an integrative statistical method for partially missing multi-omics data

Sarmistha Das[1,2] and Indranil Mukhopadhyay[1,*]

[1]Human Genetics Unit, Indian Statistical Institute, Kolkata, 700108, India
[2]Department of Biostatistics, St. Jude Children's Research Hospital, Memphis, 38105, USA
[*]indranil@isical.ac.in

Supplementary Materials

Appendix A

Lemma 1. *If $\phi(x)$ is the p.d.f. of a standard normal distribution, i.e. $\phi(x) = \frac{1}{\sqrt{2\pi}} e^{-\frac{x^2}{2}}$, $-\infty < x < \infty$, then*

$$\int_{-\infty}^{\infty} \phi\Big(\frac{\alpha x - \beta}{\sigma_1}\Big).\phi\Big(\frac{\gamma - \delta x}{\sigma_2}\Big) dx = \phi\Big(\frac{\alpha\gamma - \beta\delta}{\sqrt{\alpha^2\sigma_2^2 + \delta^2\sigma_1^2}}\Big).\frac{1}{\sqrt{\frac{\alpha^2}{\sigma_1^2} + \frac{\delta^2}{\sigma_2^2}}} \tag{1}$$

where α, β, γ, δ, σ_1^2 and σ_2^2 are constants.

Proof.

$$\begin{aligned}
&\phi\Big(\frac{\alpha x - \beta}{\sigma_1}\Big).\phi\Big(\frac{\gamma - \delta x}{\sigma_2}\Big) = \frac{1}{2\pi} \exp\Big\{ -\frac{1}{2}\Big\{\frac{(\alpha x - \beta)^2}{\sigma_1^2} + \frac{(\gamma - \delta x)^2}{\sigma_2^2}\Big\}\Big\} \\
=&\frac{1}{2\pi} \exp\Big\{ -\frac{1}{2}\Big\{\Big(\sqrt{\frac{\alpha^2}{\sigma 1^2} + \frac{\delta^2}{\sigma_2^2}}\Big(x - \frac{\alpha\beta\sigma_2^2 + \gamma\delta\sigma_1^2}{\alpha^2\sigma_2^2 + \delta^2\sigma_1^2}\Big)\Big)^2 + \Big(\frac{\alpha\gamma - \beta\delta}{\sqrt{\alpha^2\sigma_2^2 + \delta^2\sigma_1^2}}\Big)^2\Big\}\Big\} \\
=&\phi\Big(\sqrt{\frac{\alpha^2}{\sigma_1^2} + \frac{\delta^2}{\sigma_2^2}}\Big(x - \frac{\alpha\beta\sigma_2^2 + \gamma\delta\sigma_1^2}{\alpha^2\sigma_2^2 + \delta^2\sigma_1^2}\Big)\Big).\phi\Big(\frac{\alpha\gamma - \beta\delta}{\sqrt{\alpha^2\sigma_2^2 + \delta^2\sigma_1^2}}\Big)
\end{aligned} \tag{2}$$

Now integrating both sides of (6) with respect to x over the range $(-\infty, \infty)$, we have,

$$\begin{aligned}
&\int_{-\infty}^{\infty} \phi\Big(\frac{\alpha x - \beta}{\sigma_1}\Big).\phi\Big(\frac{\gamma - \delta x}{\sigma_2}\Big) dx \\
=&\phi\Big(\frac{\alpha\gamma - \beta\delta}{\sqrt{\alpha^2\sigma_2^2 + \delta^2\sigma_1^2}}\Big) \int_{-\infty}^{\infty} \phi\Big(\sqrt{\frac{\alpha^2}{\sigma_1^2} + \frac{\delta^2}{\sigma_2^2}}\Big(x - \frac{\alpha\beta\sigma_2^2 + \gamma\delta\sigma_1^2}{\alpha^2\sigma_2^2 + \delta^2\sigma_1^2}\Big)\Big) dx \\
=&\frac{1}{\sqrt{\frac{\alpha^2}{\sigma_1^2} + \frac{\delta^2}{\sigma_2^2}}}\phi\Big(\frac{\alpha\gamma - \beta\delta}{\sqrt{\alpha^2\sigma_2^2 + \delta^2\sigma_1^2}}\Big)
\end{aligned} \tag{3}$$

□

Appendix B

Result 1. *Using the model (1-3),*

$$P(y_i|\boldsymbol{Z}_{i,o}) = \sigma\Big(\frac{y_i\beta(\beta_0\boldsymbol{\beta}'_x\boldsymbol{X}_i + \beta_g G_i + \beta_m M_i + \beta_e\mu_0)}{\sqrt{\beta^2 + y_i^2\beta_e^2 + \sigma_2^2}}\Big) \tag{4}$$

where $\mu_0 = \gamma_0 + \gamma_g G_i + \gamma_m M_i$, for each $i \in S_{-E}$, where S_{-E} be the set of n_2 individuals for whom gene expression data are not available.

Proof. We write, $\boldsymbol{Z}_i = (\boldsymbol{Z}'_{i,o}, \boldsymbol{Z}'_{i,m})'$ for all $i \in S_{-E}$ where the suffixes 'o' and 'm' denote observed and missing parts. So, here $\boldsymbol{Z}_{i,o} = (1, \boldsymbol{X}'_i, G_i, M_i)'$ and $\boldsymbol{Z}_{i,m} = E_i$ for all $i \in S_{-E}$. Note that $\mathscr{C}(S_{-E}) = n_2$ where $\mathscr{C}(A)$ denotes the cardinality of a set A.

Now for each $i \in S_{-E}$, the observations on phenotype, genotype, covariates and methylation are known but gene expression values are unknown. Hence, we have for each $i \in S_{-E}$,

$$\begin{aligned}
P(y_i|\boldsymbol{Z}_{i,o}) &= \int_{Z_{i,m}} P(y_i|\boldsymbol{Z}_{i,o}, \boldsymbol{Z}_{i,m})P(\boldsymbol{Z}_{i,m}|\boldsymbol{Z}_{i,o})dZ_{i,m} \\
&= \int_{E_i} \sigma(y_i(\beta_0 + \boldsymbol{\beta}'_x\boldsymbol{X}_i + \beta_g G_i + \beta_m M_i + \beta_e E_i))\frac{1}{\sigma_2}\phi(\frac{E_i - \gamma_0 - \gamma_g G_i - \gamma_m M_i}{\sigma_2})dE_i \\
&\approx \int_{E_i}\int_{u_i=-\infty}^{y_i(\boldsymbol{w}'\boldsymbol{Z}_i)} \phi(\frac{u}{\beta})\frac{1}{\sigma_2}\phi(\frac{E_i - \mu_0}{\sigma_2})du\, dE_i \quad \text{where, } \beta = \frac{\pi}{\sqrt{3}}{}^{1} \\
&= \int_{E_i}\int_{u_i=-\infty}^{y_i(\boldsymbol{w}'_o\boldsymbol{Z}_{i,o})} \phi(\frac{\nu + y_i\beta_e E_i}{\beta})\frac{1}{\sigma_2}\phi(\frac{(E_i - \mu_0)y_i\beta_e}{\sigma_2 y_i\beta_e})d\nu\, dE_i \quad \text{where, } u = \nu + \beta_e E_i y_i
\end{aligned}$$

Now putting $x = E_i$, $\alpha = y_i\beta_e$, $\beta = \mu_0 y_i\beta_e$, $\gamma = \nu$, $\delta = -y_i\beta_i$, $\sigma_1 = \beta$, $\sigma_2 = \sigma_2 y_i\beta_e$ in Lemma 1, we have,

$$\begin{aligned}
P(y_i|\boldsymbol{Z}_{i,o}) &= \frac{1}{\beta_e\sigma_2}\frac{1}{\sqrt{2\pi}\sqrt{\frac{y_i^2}{\beta^2} + \frac{1}{\beta_e^2\sigma_2^2}}}\int_{\nu=-\infty}^{y_i\boldsymbol{w}'_o\boldsymbol{Z}_{i,o}} e^{-\frac{1}{2}\frac{(\nu+\beta_e y_i\mu_0)^2}{\beta^2+y_i^2\beta_e^2\sigma_2^2}}d\nu \\
&= \frac{1}{\sqrt{\frac{\beta^2+y_i^2\beta_e^2\sigma_2^2}{\beta^2}}}\int_{\nu=-\infty}^{y_i\boldsymbol{w}'_o\boldsymbol{Z}_{i,o}} \phi(\frac{\nu + \beta_e y_i\mu_0}{\sqrt{\beta^2 + y_i^2\beta_e^2\sigma_2^2}})d\nu \\
&= \int_{\nu'=-\infty}^{\frac{y_i\boldsymbol{w}'_o\boldsymbol{Z}_{i,o}+y_i\beta_e\mu_0}{\lambda}} \phi(\frac{\nu'}{\beta})d\nu' \text{ where, } \nu' = \frac{\nu + \beta_e y_i\mu_0}{\lambda}\ , \lambda = \sqrt{1 + \frac{y_i^2\beta_e^2\sigma_2^2}{\beta^2}} \\
&\approx \sigma(\frac{y_i\boldsymbol{w}'_o\boldsymbol{Z}_{i,o} + y_i\beta_e\mu_0}{\lambda}) \qquad \text{[using (4.8)]} \\
&= \sigma(\frac{y_i\beta(\beta_0 + \boldsymbol{\beta}'_x\boldsymbol{X}_i + \beta_g G_i + \beta_m M_i + \beta_e\mu_0)}{\sqrt{\beta^2 + y_i^2\beta_e^2\sigma_2^2}})
\end{aligned}$$

□

Result 2. *Using the model (1-3), for each $i \in S_{-M}$,*

$$P(y_i|\boldsymbol{Z}_i) = \sigma\Big(\frac{y_i\beta(\beta_0 + \boldsymbol{\beta}'_x\boldsymbol{X}_i + \beta_g G_i + \beta_e E_i + \beta_m\frac{(\alpha_0+\alpha_g G_i)\sigma_2^2+\gamma_m(E_i-\gamma_0-\gamma_g G_i)\sigma_1^2}{\sigma_2^2+\gamma_m^2\sigma_1^2})}{\sqrt{\beta^2 + \beta_m^2\frac{\sigma_1^2\sigma_2^2}{\sigma_2^2+\gamma_m^2\sigma_1^2}}}\Big) \tag{5}$$

where S_{-M} is the set of n_3 individuals for whom no methylation data are available.

Proof. From model (1-3), we have, $E(M_i) = \alpha_0 + \alpha_g G_i$, $V(M_i) = \sigma_1^2$. Now,

$$E(E_i) = E_{M_i} E_{E_i|M_i}(E_i|M_i) = E_{M_i}(\gamma_0 + \gamma_g G_i + \gamma_m M_i) = \gamma_0 + \gamma_g G_i + \gamma_m(\alpha_0 + \alpha_g G_i)$$
$$V(E_i) = E_{M_i} V_{E_i|M_i}(E_i|M_i) + V_{M_i} E_{E_i|M_i}(E_i|M_i) = \sigma_2^2 + V_{M_i}(\gamma_0 + \gamma_g G_i + \gamma_m M_i) = \sigma_2^2 + \gamma_m^2 \sigma_1^2$$
$$Cov(M_i, E_i) = Cov(M_i, \gamma_0 + \gamma_g G_i + \gamma_m M_i + \epsilon_{2i}) = \gamma_m V(M_i) = \gamma_m \sigma_1^2$$
$$\rho_{M_i,E_i} = \frac{Cov(M_i, E_i)}{\sqrt{V(E_i)V(M_i)}} = \frac{\gamma_m \sigma_1^2}{\sqrt{\sigma_1^2(\sigma_2^2 + \gamma_m^2 \sigma_1^2)}} = \frac{\gamma_m \sigma_1}{\sqrt{\sigma_2^2 + \gamma_m^2 \sigma_1^2}}$$

Hence, $P(E_i|M_i) = \frac{1}{\sigma_2}\phi(\frac{E_i - \gamma_0 - \gamma_g G_i - \gamma_m M_i - \boldsymbol{\gamma}_x' \boldsymbol{X}_i}{\sigma_2})$.
Now, for each $i \in S_{-M}$,

$$P(y_i|\boldsymbol{Z}_{i,o}) = \int_{Z_{i,m}} P(y_i|\boldsymbol{Z}_{i,o}, \boldsymbol{Z}_{i,m}) P(\boldsymbol{Z}_{i,m}|\boldsymbol{Z}_{i,o}) dZ_{i,m}$$
$$= \int_{M_i=-\infty}^{\infty} \sigma(y_i(\boldsymbol{w}'\boldsymbol{Z}_i)) P(M_i|E_i) dM_i = \int_{M_i=-\infty}^{\infty} \sigma(y_i(\boldsymbol{w}'\boldsymbol{Z}_i)) \frac{P(E_i|M_i)P(M_i)}{P(E_i)} dM_i \quad (6)$$

Now, Denominator in (12) is

$$P(E_i) = \int_{M_i} P(E_i, M_i) dM_i = \int_{M_i} P(E_i|M_i) P(M_i) dM_i$$
$$= \frac{1}{\sigma_1 \sigma_2} \int_{M_i} \phi\Big(\frac{E_i - \gamma_0 - \gamma_g G_i - \gamma_m M_i}{\sigma_2}\Big) \phi\Big(\frac{M_i - \alpha_0 - \alpha_g G_i}{\sigma_1}\Big) dM_i$$

Now in Lemma 1, put $\alpha = 1$, $\beta = \alpha_0 + \alpha_g G_i$, $\sigma_1 = \sigma_1$, $\gamma = E_i - \gamma_0 - \gamma_g G_i$, $\delta = \gamma_m$, $\sigma_2 = \sigma_2$, and noting that $\phi(-x) = \phi(x)$, we have,

$$P(E_i) = \frac{1}{\sigma_1 \sigma_2} \frac{1}{\sqrt{\frac{1}{\sigma_1^2} + \frac{\gamma_m^2}{\sigma_2^2}}} . \phi\bigg(\frac{E_i - \gamma_0 - \gamma_g G_i - \gamma_m(\alpha_0 + \alpha_g G_i)}{\sqrt{\sigma_2^2 + \gamma_m^2 \sigma_1^2}}\bigg)$$
$$= \frac{1}{\sqrt{\sigma_2^2 + \gamma_m^2 \sigma_1^2}} . \phi\bigg(\frac{a\gamma_m - b}{\sqrt{\sigma_2^2 + \gamma_m^2 \sigma_1^2}}\bigg) \quad (7)$$

where $a = \alpha_0 + \alpha_g G_i$ and $b = E_i - \gamma_0 - \gamma_g G_i$

Now putting $p = \frac{a\sigma_2^2 + \gamma_m b \sigma_1^2}{\sigma_2^2 + \gamma_m^2 \sigma_1^2}$, $q = \frac{1}{\sqrt{\frac{1}{\sigma_1^2} + \frac{\gamma_m^2}{\sigma_2^2}}} = \frac{\sigma_1 \sigma_2}{\sigma_2^2 + \gamma_m^2 \sigma_1^2}$ and using Lemma 1 and (8), we can simplify the numerator

of (12) as:

$$
\begin{aligned}
&\int_{M_i=-\infty}^{\infty} \sigma(y_i(\boldsymbol{w}'\boldsymbol{Z}_i))P(M_i|E_i)P(M_i)dM_i \\
&= \frac{1}{\sigma_1\sigma_2}\int_{M_i} \sigma(y_i\boldsymbol{w}'\boldsymbol{Z}_i)\phi\Big(\frac{E_i-\gamma_0-\gamma_g G_i-\gamma_m M_i}{\sigma_2}\Big)\phi\Big(\frac{M_i-\alpha_0-\alpha_g G_i}{\sigma_1}\Big)dM_i \\
&= \frac{1}{\sigma_1\sigma_2}\int_{M_i} \sigma(y_i\boldsymbol{w}'\boldsymbol{Z}_i)\phi\Big(\frac{a\gamma_m-b}{\sqrt{\sigma_2^2+\gamma_m^2\sigma_1^2}}\Big)\phi\Big(\sqrt{\frac{1}{\sigma_1^2}+\frac{\gamma_m^2}{\sigma_2^2}}(M_i-\frac{a\sigma_2^2+\gamma_m^2 b\sigma_1^2}{\sigma_2^2+\gamma_m^2\sigma_1^2})\Big)dM_i \\
&\approx \frac{1}{\sigma_1\sigma_2}\phi(\frac{a\gamma_m-b}{\sqrt{\sigma_2^2+\gamma_m^2\sigma_1^2}})\int_{M_i}\int_{u=-\infty}^{y_i\boldsymbol{w}'\boldsymbol{Z}_i}\phi(\frac{u}{\beta})\phi(\frac{M_i-p}{q})\,du\,dM_i \\
&= \frac{1}{\sigma_1\sigma_2}\frac{1}{\beta_m}\phi(\frac{a\gamma_m-b}{\sqrt{\sigma_2^2+\gamma_m^2\sigma_1^2}})\int_{M_i^*}\int_{\nu=-\infty}^{y_i(\beta_0+\boldsymbol{\beta}_x'\boldsymbol{X}_i+\beta_g G_i+\beta_e E_i)}\phi(\frac{\nu+y_iM_i^*}{\beta})\phi(\frac{M_i^*-\beta_m p}{\beta_m q})\,d\nu\,dM_i^* \\
&\text{where } u=\nu+y_i\beta_m M_i,\ M_i^*=\beta_m M_i \\
&= \frac{\beta q}{\sigma_1\sigma_2\sqrt{\beta^2+\beta_m^2q^2}}\phi(\frac{a\gamma_m-b}{\sqrt{\sigma_2^2+\gamma_m^2\sigma_1^2}})\int_{\nu=-\infty}^{y_i(\beta_0+\boldsymbol{\beta}_x'\boldsymbol{X}_i+\beta_g G_i+\beta_e E_i)}\phi(\frac{\nu+y_i\beta_m p}{\sqrt{\beta^2+\beta_m^2q^2}})d\nu \\
&= \frac{q}{\sigma_1\sigma_2}\phi(\frac{a\gamma_m-b}{\sqrt{\sigma_2^2+\gamma_m^2\sigma_1^2}})\int_{\nu'=-\infty}^{\frac{y_i\beta(\beta_0+\boldsymbol{\beta}_x\boldsymbol{X}_i+\beta_g G_i+\beta_e E_i+\beta_m p)}{\sqrt{\beta^2+\beta_m^2q^2}}}\phi(\frac{\nu'}{\beta})d\nu',\ \text{where } \frac{\nu'}{\beta}=\frac{\nu+y_i\beta_m p}{\sqrt{\beta^2+\beta_m^2q^2}} \\
&\approx \frac{1}{\sqrt{\sigma_2^2+\gamma_m^2\sigma_1^2}}\phi(\frac{a\gamma_m-b}{\sqrt{\sigma_2^2+\gamma_m^2\sigma_1^2}})\sigma(\frac{y_i\beta(\beta_0+\boldsymbol{\beta}_x'\boldsymbol{X}_i+\beta_g G_i+\beta_e E_i+\beta_m p)}{\sqrt{\beta^2+\beta_m^2q^2}})\ \text{[using (8)]}
\end{aligned} \tag{8}
$$

Therefore, using (13) and (14) in (12), we have,

$$
\begin{aligned}
P(y_i|\boldsymbol{Z}_{i,o}) &= \int_{M_i=-\infty}^{\infty}\sigma(y_i(\boldsymbol{w}'\boldsymbol{Z}_i))\frac{P(E_i|M_i)P(M_i)}{P(E_i)}dM_i \\
&\approx \sigma\Big(\frac{y_i\beta(\beta_0+\boldsymbol{\beta}_x'\boldsymbol{X}_i+\beta_g G_i+\beta_e E_i+\beta_m p)}{\sqrt{\beta^2+\beta_m^2q^2}}\Big) \\
&= \sigma\Big(\frac{y_i\beta(\beta_0+\boldsymbol{\beta}_x'\boldsymbol{X}_i+\beta_g G_i+\beta_e E_i+\beta_m\frac{(\alpha_0+\alpha_g G_i)\sigma_2^2+\gamma_m(E_i-\gamma_0-\gamma_g G_i)\sigma_1^2}{\sigma_2^2+\gamma_m^2\sigma_1^2})}{\sqrt{\beta^2+\beta_m^2\frac{\sigma_1^2\sigma_2^2}{\sigma_2^2+\gamma_m^2\sigma_1^2}}}\Big)
\end{aligned} \tag{9}
$$

□

Result 3. *Under the model (1-3), for each $i \in S_{-(E,M)}$,*

$$
P(y_i|\boldsymbol{Z}_{i,o}) = \sigma(\frac{y_i\beta(p_1(\beta_0+\boldsymbol{\beta}_xX_i+\beta_g G_i)+\beta_m a\sigma_2^2-\beta_m\gamma_m\sigma_1^2(\gamma_0+\gamma_g G_i)+\delta_3)}{\sqrt{\delta_2^2+\delta_4^2}}) \tag{10}
$$

where $S_{-(E,M)}$ is the set of n_4 individuals for whom both expression and methylation data are missing.

Proof. For each $i \in S_{-(E,M)}$,

$$
\begin{aligned}
P(y_i|\boldsymbol{Z}_{i,o}) &= \int\limits_{Z_{i,m}} P(y_i|\boldsymbol{Z}_{i,o},\boldsymbol{Z}_{i,m})P(\boldsymbol{Z}_{i,m}|\boldsymbol{Z}_{i,o})\, dZ_{i,m} \\
&= \int\limits_{M_i=-\infty}^{\infty}\int\limits_{E_i=-\infty}^{\infty} \sigma(y_i(\boldsymbol{w}'\boldsymbol{Z}_i))P(E_i|M_i)P(M_i)\, dE_i\, dM_i \\
&= \int\limits_{M_i=-\infty}^{\infty}\int\limits_{E_i=-\infty}^{\infty} \Big\{\sigma(y_i(\boldsymbol{w}'\boldsymbol{Z}_i))\frac{1}{\sigma_2}\phi(\frac{E_i-\gamma_0-\gamma_g G_i-\gamma_m M_i-\boldsymbol{\gamma}_x'\boldsymbol{X}_i}{\sigma_2}) \\
&\qquad \times \frac{1}{\sigma_1}\phi(\frac{M_i-\alpha_0-\alpha_g G_i-\boldsymbol{\alpha}_x'\boldsymbol{X}_i}{\sigma_1})\Big\} dE_i dM_i \\
&\approx \int\limits_{M_i=-\infty}^{\infty}\int\limits_{E_i=-\infty}^{\infty}\int\limits_{u=-\infty}^{y_i\boldsymbol{w}'\boldsymbol{Z}_i} \phi(\frac{u}{\beta})\frac{1}{\sigma_2}\phi(\frac{E_i-\gamma_0-\gamma_g G_i-\gamma_m M_i}{\sigma_2})\frac{1}{\sigma_1}\phi(\frac{M_i-\alpha_0-\alpha_g G_i}{\sigma_1}) du dE_i dM_i \\
&\text{where, } \beta = \frac{\pi}{\sqrt{3}}\,[1] \\
&= \int\limits_{M_i}\int\limits_{E_i}\int\limits_{\nu=-\infty}^{y_i(\beta_0+\boldsymbol{\beta}_x' X_i+\beta_g G_i)} \Big\{\frac{1}{\sigma_1\sigma_2}\phi(\frac{\nu+y_i\beta_e E_i+y_i\beta_m M_i}{\beta})\phi(\frac{E_i-\gamma_0-\gamma_g G_i-\gamma_m M_i}{\sigma_2}) \\
&\qquad \times \phi(\frac{M_i-\alpha_0-\alpha_g G_i}{\sigma_1})\Big\}\, d\nu\, dE_i\, dM_i, \text{ where, } u = \nu+y_i\beta_e E_i+y_i\beta_m M_i \qquad (11)
\end{aligned}
$$

Now in Lemma 1, put $x = E_i$, $\alpha = 1$, $\beta = \gamma_0 + \gamma_g G_i + \gamma_m M_i$, $\sigma_1 = \sigma_2$, $\gamma = \nu + y_i\beta_m M_i$, $\delta = -y_i\beta_e$, and $\sigma_2 = \beta = \frac{\pi}{\sqrt{3}}$, to get,

$$
\begin{aligned}
&\int\limits_{E_i=-\infty}^{\infty} \phi(\frac{\nu+y_i\beta_e E_i+y_i\beta_m M_i}{\beta})\phi(\frac{E_i-\gamma_0-\gamma_g G_i-\gamma_m M_i}{\sigma_2})\, dE_i \\
&= \frac{1}{\sqrt{\frac{1}{\sigma_2^2}+\frac{y_i^2\beta_e^2}{\beta^2}}}\phi(\frac{\nu+y_i\beta_m M_i+y_i\beta_e(\gamma_0+\gamma_g G_i+\gamma_m M_i)}{\sqrt{\beta^2+y_i^2\beta_e^2\sigma_2^2}}) \qquad (12)
\end{aligned}
$$

Again putting $x = M_i$, $\alpha = 1$, $\beta = \alpha_0 + \alpha_g G_i$, $\sigma_1 = \sigma_1$, $\gamma = \nu + y_i\beta_e(\gamma_0 + \gamma_g G_i)$, $\delta = -(y_i\beta_e\gamma_m + y_i\beta_m$, $\sigma_2 = \sqrt{\beta^2 + y_i^2\beta_e^2\sigma_2^2}$, we have,

$$\frac{1}{\sqrt{\frac{1}{\sigma_2^2}+\frac{y_i^2\beta_e^2}{\beta^2}}}\int_{M_i=-\infty}^{\infty}\Big\{\phi(\frac{M_i-\alpha_0-\alpha_h G_i}{\sigma_1}$$

$$\times\,\phi(\frac{\nu+y_i\beta_m M_i+y_i\beta_e(\gamma_0+\gamma_g G_i+\gamma_m M_i)}{\sqrt{\beta^2+y_i^2\beta_e^2\sigma_2^2}})\Big\}\,dM_i$$

$$=\frac{1}{\sqrt{\frac{1}{\sigma_2^2}+\frac{y_i^2\beta_e^2}{\beta^2}}}\frac{1}{\sqrt{\frac{1}{\sigma_1^2}+\frac{y_i^2(\beta_e\gamma_m+\beta_m)^2}{\beta^2+y_i^2\beta_e^2\sigma_2^2}}}$$

$$\times\,\phi(\frac{\nu+y_i\beta_e(\gamma_0+\gamma_g G_i)+(\alpha_0+\alpha_g G_i)(y_i\beta_e\gamma_m+y_i\beta_m)}{\sqrt{\beta^2+y_i^2\beta_e^2\sigma_2^2+y_i^2(\beta_e\gamma_m+\beta_m)^2\sigma_1^2}})$$

$$=\frac{1}{\sqrt{\frac{1}{\sigma_2^2}+\frac{y_i^2\beta_e^2}{\beta^2}}}\frac{1}{\sqrt{\frac{1}{\sigma_1^2}+\frac{y_i^2(\beta_e\gamma_m+\beta_m)^2}{\beta^2+y_i^2\beta_e^2\sigma_2^2}}}\phi(\frac{\nu+y_i\beta_e d+a(y_i\beta_e\gamma_m+y_i\beta_m)}{\sqrt{\beta^2+y_i^2\beta_e^2\sigma_2^2+y_i^2(\beta_e\gamma_m+\beta_m)^2\sigma_1^2}})$$

$$=\frac{\beta\sigma_1\sigma_2}{\delta_1}\phi(\frac{\nu+y_i\beta_e d+a(y_i\beta_e\gamma_m+y_i\beta_m)}{\delta_1}) \tag{13}$$

where $a=(\alpha_0+\alpha_g G_i)$, $d=(\gamma_0+\gamma_g G_i)$ and $\delta_1^2=\beta^2+y_i^2\beta_e^2\sigma_2^2+y_i^2(\beta_e\gamma_m+\beta_m)^2\sigma_1^2$.

$$P(y_i|\boldsymbol{Z}_{i,o})=\int_{\nu=-\infty}^{y_i\boldsymbol{w}_o'\boldsymbol{Z}_{i,o}}\frac{1}{\sigma_1\sigma_2}\frac{\beta\sigma_1\sigma_2}{\delta_1}\phi(\frac{\nu+y_i\beta_e d+a(y_i\beta_e\gamma_m+y_i\beta_m)}{\delta_1})\,d\nu$$

$$=\sigma(\frac{\beta y_i(\beta_0+\beta_g G_i+\boldsymbol{\beta}_x'\boldsymbol{X}_i+\beta_e(\gamma_0+\gamma_g G_i)+(\alpha_0+\alpha_g G_i)(\beta_e\gamma_m+\beta_m))}{\sqrt{\beta^2+y_i^2\beta_e^2\sigma_2^2+y_i^2(\beta_e\gamma_m+\beta_m)^2\sigma_1^2}}) \tag{14}$$

□

Table S1: Type I Error under different combination of sample sizes and varying percentages of missing methylation and/or gene expression values based on 10000 simulations

SS / *missing %*	100		150		200		*SS* / *missing %*	100		150		200	
	KNN	MI	KNN	MI	KNN	MI		KNN	MI	KNN	MI	KNN	MI
(0,0,0)	0.0534	0.0534	0.0528	0.0528	0.0533	0.0533	(10,0,10)	0.0566	0.1217	0.0489	0.0929	0.0471	0.0785
(0,0,10)	0.0546	0.0885	0.0541	0.0722	0.0536	0.0624	(0,10,10)	0.0566	0.0875	0.0548	0.0732	0.0524	0.0679
(0,0,20)	0.0535	0.1423	0.055	0.1031	0.0516	0.0842	(10,10,0)	0.0595	0.1222	0.0479	0.0950	0.0464	0.0829
(0,0,40)	0.0505	0.3331	0.0521	0.1919	0.0511	0.1406	(20,0,20)	0.0451	0.2654	0.0437	0.1592	0.0397	0.1159
(0,0,60)	0.0552	0.6094	0.0503	0.3168	0.0502	0.2208	(0,20,20)	0.0453	0.1466	0.0476	0.1050	0.0441	0.0868
(0,0,80)	0.0391	0.6104	0.0425	0.3173	0.0424	0.2192	(20,20,0)	0.0444	0.2578	0.0452	0.1535	0.0389	0.1243
(0,10,0)	0.0591	0.0733	0.0543	0.0685	0.0560	0.0607	(40,0,40)	0.0218	0.7302	0.0209	0.3844	0.0240	0.2518
(0,20,0)	0.0503	0.1030	0.0474	0.0858	0.0481	0.0731	(0,40,40)	0.0321	0.3449	0.0330	0.1944	0.0333	0.1344
(0,40,0)	0.0367	0.2166	0.0357	0.1373	0.037	0.1053	(10,10,10)	0.0457	0.1447	0.0452	0.1040	0.0433	0.0887
(0,60,0)	0.0310	0.4110	0.0317	0.2171	0.0307	0.1591	(10,20,10)	0.0367	0.2214	0.0372	0.1414	0.0389	0.1143
(0,80,0)	0.0277	0.6625	0.0296	0.3240	0.0271	0.2209	(10,10,20)	0.0474	0.1799	0.0429	0.1216	0.0451	0.0981
(10,0,0)	0.0582	0.0737	0.0506	0.0680	0.0520	0.0595	(20,10,10)	0.0372	0.2299	0.0374	0.1400	0.0349	0.1091
(20,0,0)	0.0442	0.1060	0.0398	0.0927	0.0401	0.0705	(20,10,20)	0.0392	0.2822	0.0362	0.1630	0.0371	0.1295
(40,0,0)	0.0315	0.2132	0.0314	0.1346	0.0308	0.1148	(20,20,10)	0.0336	0.2906	0.0322	0.1702	0.0339	0.1258
(60,0,0)	0.0290	0.4069	0.0258	0.2191	0.0244	0.1500	(10,20,20)	0.0413	0.2282	0.0331	0.1377	0.0357	0.1101
(80,0,0)	0.0173	0.6716	0.0164	0.3298	0.0204	0.2105	(20,20,20)	0.0358	0.3386	0.0308	0.1892	0.0330	0.1377

missing% $(m_1, m_2, m_3) \equiv$ (m_1% both missing, m_2% only methylation missing, m_3% only gene expression missing); *SS*: sample size for case (or control)

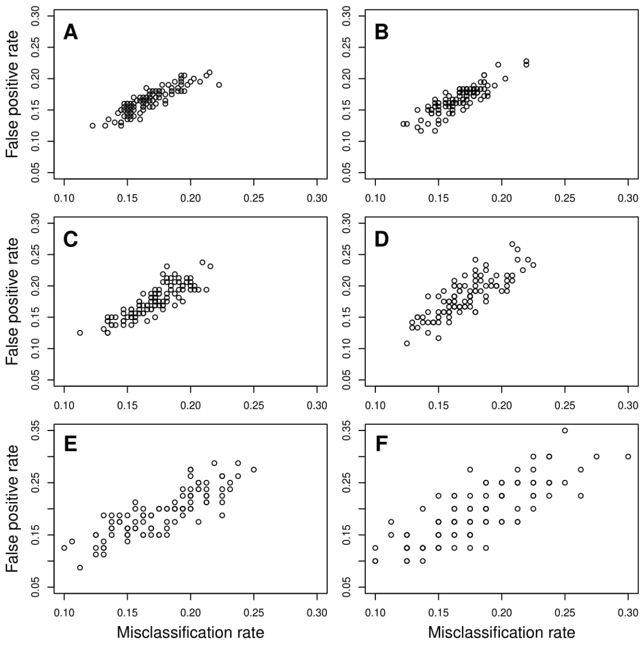

Figure $S1$: Plot of Misclassification rate vs False positive rate (1-Specificity) for only methylation missing. Figure S1A depicts no missing data scenario while Figures S1B-S1F respectively depict 10%, 20%, 40%, 60% and 80% only methylation data missing scenarios

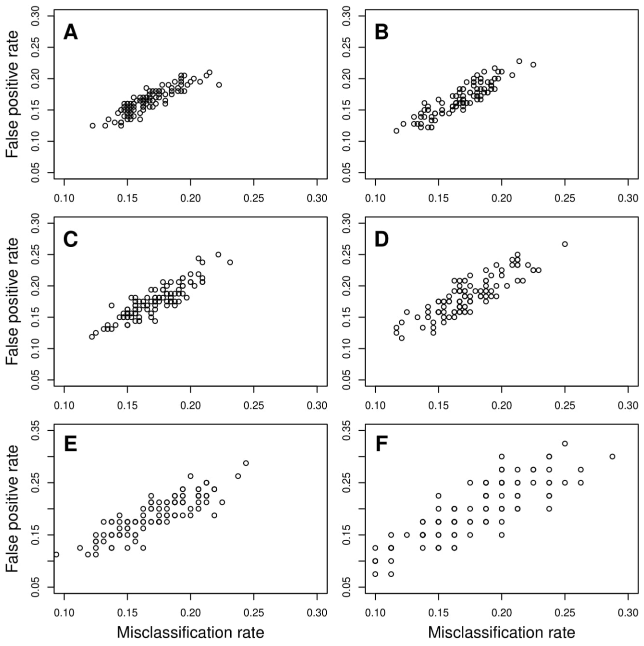

Figure $S2$: Plot of Misclassification rate vs False positive rate (1-Specificity) for both missing. Figure S2A depicts no missing data scenario while Figures S2B-S2F respectively depict 10%, 20%, 40%, 60% and 80% of both gene expression and methylation data missing scenarios

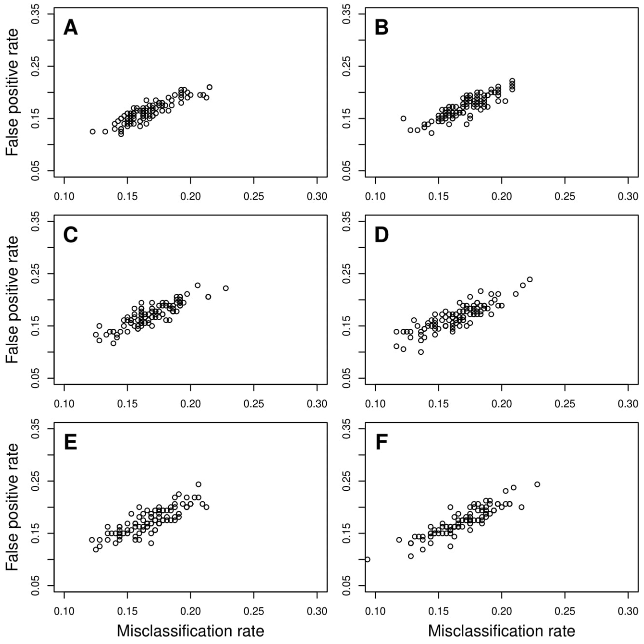

Figure $S3$: Plot of Misclassification rate vs False positive rate (1-Specificity) for miscellaneous missing. Figure S3A depicts no missing data scenario; Figure S3B depicts 5% individuals with methylation missing and another 5% with both gene expression and methylation missing but none of the individuals have only gene expression missing; similarly in Figure S3C the percentages of missing only methylation, both omics and only gene expression are respectively 0%, 5%, 5%; in Figures S3D-S3F these percentages are (5,0,5), (10,10,0), (5,10,5) respectively.

Table S2: Power under different combination of sample sizes and varying percentages of missing methylation and/or gene expression values based on 1000 simulations

SS / *missing %*	100		150		200		*SS* / *missing %*	100		150		200	
	KNN	MI	KNN	MI	KNN	MI		KNN	MI	KNN	MI	KNN	MI
(0,0,0)	0.697	0.697	0.878	0.878	0.950	0.950	(10,0,10)	0.454	0.508	0.638	0.767	0.791	0.871
(0,0,10)	0.524	0.547	0.707	0.781	0.837	0.890	(0,10,10)	0.518	0.563	0.695	0.750	0.847	0.893
(0,0,20)	0.478	0.490	0.667	0.717	0.827	0.870	(10,10,0)	0.469	0.461	0.712	0.729	0.851	0.889
(0,0,40)	0.467	0.401	0.679	0.655	0.804	0.822	(20,0,20)	0.351	0.369	0.581	0.633	0.736	0.831
(0,0,60)	0.460	0.282	0.692	0.565	0.819	0.768	(0,20,20)	0.502	0.503	0.662	0.728	0.822	0.865
(0,0,80)	0.517	0.162	0.707	0.463	0.820	0.712	(20,20,0)	0.447	0.347	0.637	0.668	0.788	0.820
(0,10,0)	0.561	0.537	0.795	0.761	0.890	0.913	(40,0,40)	0.398	0.169	0.542	0.410	0.664	0.678
(0,20,0)	0.576	0.518	0.826	0.767	0.892	0.890	(0,40,40)	0.456	0.554	0.578	0.704	0.795	0.813
(0,40,0)	0.662	0.359	0.832	0.660	0.924	0.862	(10,10,10)	0.471	0.494	0.667	0.714	0.811	0.845
(0,60,0)	0.643	0.229	0.832	0.538	0.946	0.783	(10,20,10)	0.517	0.408	0.673	0.665	0.813	0.822
(0,80,0)	0.698	0.134	0.855	0.405	0.948	0.705	(10,10,20)	0.446	0.474	0.605	0.682	0.762	0.838
(10,0,0)	0.536	0.610	0.692	0.822	0.838	0.928	(20,10,10)	0.432	0.429	0.621	0.638	0.778	0.818
(20,0,0)	0.464	0.565	0.653	0.786	0.794	0.925	(20,10,20)	0.432	0.386	0.586	0.625	0.711	0.819
(40,0,0)	0.424	0.432	0.568	0.695	0.706	0.858	(20,20,10)	0.435	0.356	0.644	0.626	0.752	0.824
(60,0,0)	0.361	0.278	0.559	0.635	0.686	0.801	(10,20,20)	0.461	0.446	0.665	0.642	0.800	0.832
(80,0,0)	0.378	0.275	0.493	0.632	0.659	0.801	(20,20,20)	0.396	0.376	0.594	0.625	0.732	0.816

missing% $(m_1, m_2, m_3) \equiv$ (m_1% both missing, m_2% only methylation missing, m_3% only gene expression missing); *SS*: sample size for case (or control)

Table $S3$: Mean (±standard deviation) of prediction accuracy, specificity, sensitivity based on 10-fold cross validation of 100 datasets for each pre-assigned missing omics data structure

missing %	Prediction accuracy	Specificity	Sensitivity
(0,0,0)	83.275 (±1.954)	0.836 (±0.020)	0.822 (±0.024)
(0,0,10)	83.464 (±1.839)	0.835 (±0.022)	0.834 (±0.022)
(0,0,20)	83.406 (±1.993)	0.833 (±0.024)	0.833 (±0.024)
(0,0,40)	83.167 (±2.738)	0.825 (±0.034)	0.839 (±0.030)
(0,0,60)	82.775 (±3.040)	0.815 (±0.042)	0.841 (±0.039)
(0,0,80)	82.537 (±4.429)	0.815 (±0.059)	0.835 (±0.057)
(0,10,0)	83.531 (±1.773)	0.833 (±0.021)	0.838 (±0.022)
(0,20,0)	83.109 (±2.204)	0.827 (±0.027)	0.835 (±0.024)
(0,40,0)	83.133 (±2.259)	0.820 (±0.030)	0.843 (±0.026)
(0,60,0)	81.944 (±3.339)	0.805 (±0.041)	0.833 (±0.039)
(0,80,0)	82.500 (±4.533)	0.813 (±0.059)	0.837 (±0.058)
(10,0,0)	83.272 (±2.274)	0.832 (±0.026)	0.833 (±0.025)
(20,0,0)	83.087 (±1.970)	0.827 (±0.023)	0.834 (±0.021)
(40,0,0)	82.800 (±2.546)	0.820 (±0.031)	0.836 (±0.029)
(60,0,0)	82.687 (±3.070)	0.809 (±0.039)	0.844 (±0.034)
(80,0,0)	82.712 (±4.619)	0.808 (±0.058)	0.847 (±0.051)
(5,0,5)	83.092 (±1.892)	0.828 (±0.021)	0.833 (±0.022)
(0,5,5)	83.405 (±2.068)	0.831 (±0.023)	0.837 (±0.025)
(5,5,0)	82.967 (±1.828)	0.828 (±0.022)	0.831 (±0.021)
(10,0,10)	83.303 (±2.184)	0.829 (±0.027)	0.837 (±0.025)
(10,10,0)	82.991 (±2.134)	0.825 (±0.026)	0.835 (±0.025)
(10,5,5)	83.444 (±2.361)	0.831 (±0.028)	0.838 (±0.025)

missing% $(m_1, m_2, m_3) \equiv$ (m_1% both missing, m_2% only methylation missing, m_3% only expression missing)

Table S4: List of significant TiMEG genes associated with TSC disease

ACACA	ACSS2	ACTR3C	AKAP12	ALDH1L1	AMOTL2	ANKRD36B	ANO7	APP	ARHGEF16
ARSG	ARTN	ASAP1	ASPA	ASPRV1	ATG9B	ATP1B2	ATP6V1C2	BCOR	BEND6
C1GALT1	C3orf35	C3orf67	C4orf19	C4orf3	C5orf22	C8orf31	CASQ1	CBFA2T2	CCDC149
CCDC7	CEBPG	CHST10	CHST13	CLCC1	CLDN15	CNGB3	CNTFR	CNTNAP1	CPD
CRB2	CREB5	CRIM1	CSNK1G2	CUL9	DCBLD1	DCP2	DLX1	DLX3	DNAH17
DPY19L1	DPYSL5	DRD4	ECT2L	EFCAB1	EFNA2	ELOVL7	EMX2OS	EPDR1	ERCC6
EXOC2	FAAH2	FAM110A	FGD5	FGFR2	FHDC1	FOXN2	FSCN2	GNG4	GPM6A
HIF3A	HIST1H4C	HTR2C	HTT	IL1B	IQCE	JAK3	KANK3	KCND3	KCNQ1OT1
KIAA1109	KLHDC8A	KLHL18	LAMC2	LANCL2	LDLRAP1	LIFR	LLGL2	LMNB1	LOC100270746
LOC407835	LOC441455	LOC730101	LOC90246	LRP1B	MAFF	MAGI3	MAP4K4	MMRN2	MORN1
MYH10	MYH15	MYLK2	NAV1	NCK2	NEBL	NIPSNAP3B	NLRP14	NMT1	NOLC1
NPR3	NT5M	OBSCN	PCBP4	PMEPA1	PRKG1	PRRT4	PSAT1	PTK6	PTPDC1
RAP2C	RBMS3	RESP18	RNF103	RNF207	RRP15	SEL1L3	SERPINF1	SGSM2	SIPA1L3
SLC16A8	SLC27A6	SLC29A1	SLC7A11	SMAD7	SMPDL3B	SMTNL2	SNTG2	SNX31	SPRY1
SPTBN1	SRI	SRPK3	STK25	STX11	SULF2	TACO1	TBC1D12	TBC1D14	TBX1
TCEA2	TG	THSD7A	TOX2	TRAPPC2	TRAPPC9	TSC1	TSC22D4	USH2A	VILL
VIPR2	VPS13D	WDR27	WIPF1	WNT2B	WWC2	ZDHHC11	ZNF239	ZNF275	ZNF876P

References

1. Williams, D., Liao, X., Xue, Y., and Carin, L. (2005). Incomplete-data classification using logistic regression. In Proceedings of the 22nd International Conference on Machine learning (ACM), pp. 972–979.
